# Transmission-type photonic doping for high-efficiency epsilon-near-zero supercoupling

**DOI:** 10.1038/s41467-023-41965-5

**Published:** 2023-10-03

**Authors:** Wendi Yan, Ziheng Zhou, Hao Li, Yue Li

**Affiliations:** 1 Department of Electronic Engineering, Tsinghua University, 100084 Beijing, China; 2 College of Physics and Information Engineering, Fuzhou University, 350108 Fuzhou, China; 3 Beijing National Research Center for Information Science and Technology, 100084 Beijing, China

**Keywords:** Photonic devices, Metamaterials, Electrical and electronic engineering

## Abstract

Supercoupling effect is an exotic and counterintuitive physical phenomenon of epsilon-near-zero (ENZ) media, in which the light can be “squeezed” and tunneled through flexible channels substantially narrower than its wavelength. Theoretically, ENZ channels with infinitely small widths perform ideal supercoupling with full energy transmission and zero-phase advance. As a feasible solution to demonstrate ENZ supercoupling through a finite-width channel, photonic doping can assist the light in squeezing, but the resonant dopant introduces inevitable losses. Here, we propose an approach of transmission-type photonic doping to achieve proximate ideal ENZ supercoupling. In contrast to the conventional resonance-type photonic doping, our proposed transmission-type doping replaces high-quality-factor two-dimensional resonant doping modes with low-quality-factor one-dimensional modes, such that obviously high transmission efficiency and zero-phase advance in ENZ supercoupling is achieved and observed in experiments. Benefiting from the high-efficiency ENZ supercoupling, waveguides with near-total energy transmission can be engineered with arbitrary dimensions and shapes, serving as flexible power conduits in the paradigm of waveguide integrated circuits for future millimeter-wave and terahertz integrated circuit innovations.

## Abstract (web summary)

The authors present a transmission-type doping approach to reduce resonant losses in photonic doping. Assisted by the approach, proximate ideal epsilon-near-zero (ENZ) supercoupling with neartotal energy transmission and zero-phase advance is achieved in experiments.

## Introduction

Media with extremely small permittivity, i.e., the epsilon-near-zero (ENZ) media^1–3^, have attracted considerable interest from researchers in the fields of physics, material science, and electromagnetism recently, due to their novel electromagnetic (EM) properties. EM waves in ENZ media feature stretched wavelengths much larger than those in free space, thereby breaking the constraints of the structural size of conventional periodic metamaterials and metasurfaces^4–6^. Exotic wave phenomena and functionalities including supercoupling^7–10^, wavefront transformation^11,12^, enhancement of optical nonlinearity^13,14^, photonic traps in open structures^15,16^, and ideal EM power flow^17,18^ have been investigated by researchers. Among them, the most notable one is supercoupling, which is defined as the capability of EM waves to be “squeezed” and tunneled through channels much narrower than their wavelengths^7,8^. The concept of supercoupling not only extends the mechanism of tunneling from the field of quantum mechanics to electromagnetism but also explores the possibility of propagating EM waves at frequencies below the cutoff frequency using ultranarrow waveguides^9,10^. In addition, ENZ supercoupling demonstrates extraordinary potentials in enabling EM waves to overcome the sharp bends^8^, corners^19^, and obstacles^17^. Supercoupling allows for reflectionless propagation in arbitrary-shaped waveguides^19–21^, constructing multiple applications including enhanced plasmonic channels^22^, coaxial-to-waveguide matchers^23^ and electrical-plane (E-plane) electric fibers^24^, to name just a few. However, according to the theoretical prediction^7,8^, the ideal supercoupling, i.e., lossless EM waves transmission with zero-phase shift and obvious slow light effect requires infinitely narrow waveguides filled with lossless ENZ media, which is infeasible in practical applications. ENZ channels with finite widths bring undesirable loss, thereby limiting the potential applications of supercoupling^9,10^. Another mechanism for achieving total energy transmission in waveguides is the resonant coupling, which can occur in various background media and mitigate losses caused by finite-width channels^25–27^. However, the resonant coupling effect requires symmetrical waveguide structures and is difficult to repeat the unique performance of supercoupling in sharp bends, corners, and obstacles. Consequently, there exists an urgent demand for a highly efficient ENZ supercoupling solution that can be effectively applied in engineering contexts.

Benefiting from the ability to modulate the equivalent EM characteristics in a deep subwavelength scale, ENZ metamaterials provide a platform for manipulating wave-matter interactions as well as realizing a batch of novel metamaterial devices^24,28–32^. Photonic doping^28^, a viable implementation of ENZ metamaterials, is accomplished by locating dielectric rods with high permittivity inside the ENZ media, providing a prospective method to tune effective permeability, thereby manipulating the intrinsic impedances as well as the electromagnetic scattering behaviors of the ENZ systems. Recently, various applications based on photonic-doping ENZ metamaterials including substrate-integrated waveguide (SIW) devices^24^, impedance matchers^29^, geometry-independent antennas^30^, and calculus calculators^31^ have been investigated, illustrating the enormous application opportunities for ENZ metamaterials. According to the theoretical prediction of ideal photonic doping, the tuning range of the efficient permeability can change from negative infinity to positive infinity^28^, attaining two specific cases including epsilon-and-mu-near-zero (EMNZ) media^33–35^ with a near-zero permeability and perfect magnetic conductor (PMC) body with an infinite permeability. The ideal EMNZ media (without any material loss) enables full energy transmission of EM waves with zero-phase shift. Therefore, we can enhance the transmission efficiency of supercoupling through constructing EMNZ states in ENZ channel. However, in practice, resonances in lossy ENZ and dopants consume considerable amount of energy, inevitably reducing the efficiency of supercoupling^24,28,32^. The efficiency of ENZ supercoupling using conventional photonic doping, i.e., resonance-type doping to match impedance has no significant improvement compared to the narrow channel case^9,10,24^. In order to maximize the transmission efficiency of supercoupling, it is also necessary to find a new methodology that can decrease the resonance losses in photonic doping as much as possible.

In this work, we propose a new type of photonic doping named as “transmission-type doping”, which enhances the transmission efficiency of ENZ supercoupling. We illustrate that transmission-type doping performs a different physical mechanism through a comparison of EM power flow with that of traditional resonance-type doping^17,18^. Based on the transmission line theory^36^, we demonstrate that the proposed transmission-type doping supports low-quality-factor (low-Q) one-dimensional (1D) modes inside the dopant. In contrast to high-quality-factor (high-Q) two-dimensional (2D) resonant modes in resonance-type doping, the proposed configuration relieves the intrinsic losses introduced by the dielectric dopant, ultimately achieving the high-efficiency ENZ supercoupling. This high-efficiency method of EM wave propagation, assisted by transmission-type doping, inherits the favorable characteristics of ENZ supercoupling^8,17,19^, including achieving full transmission with zero-phase advance through distorted waveguides with bends, corners and obstacles. However, because of the specific modes in transmission-type doping, the transmission amplitude and phase are position-dependent on the dopant. With the assistance of waveguide-emulated plasma^37,38^, we theoretically design and experimentally verify a three-dimensional (3D) configuration for high-efficiency ENZ supercoupling. The measurement results demonstrate that, under the condition of zero-phase shift, we realize an enhanced transmission efficiency of at least 20% higher compared to the original works^9,10,24^, thereby achieving a record-breaking efficiency of ENZ supercoupling in experiments. We also experimentally verify the supercoupling characteristics in the 3D configuration as another evidence for high-efficiency ENZ supercoupling. Benefiting from both high-efficiency transmission and the characteristics of ENZ supercoupling, we design “H-plane electric fibers”. These fibers allow EM waves to overcome the enormous discontinuity in the magnetic plane (H-plane) and propagate in waveguides of arbitrary shapes at a subwavelength scale. Compared to E-plane electric fibers^24^, H-plane electric fibers offer three irreplaceable advantages. First, they demonstrate a significant enhancement in transmission efficiency. Second, they are not limited by the profile height of waveguides, allowing for placement in ultrathin scenarios. Third, excluding height skipping of the E-plane electric fibers^24^, H-plane electric fibers facilitate the integration of various techniques in engineering fabrication considerations^39,40^. It is well known that waveguides prevent energy leakage more effectively than microstrip transmission lines for millimeter-wave and terahertz frequencies^41,42^ and are thereby favorable competitive solutions for the next generation integrated devices^43,44^. We confirm that our work realizes low-loss and flexible power conduits in future waveguide-integrated architectures, which have great significance for the development of metamaterial-based millimeter-wave and terahertz integrated circuit innovations.

## Results

### General concept

We begin the discussion with the general ENZ supercoupling model, which consists of an arbitrary-shaped ENZ channel with the area *A*_p_ connecting two rectangular waveguide ports with the widths of *a*_1_ and *a*_2_^7,8^. The EM waves are excited from Port 1, squeezed through the ENZ channel, and received at Port 2. As demonstrated theoretically, the reflection coefficient can be described as^7^

1 $$\rho=\frac{({a}_{1}-{a}_{2})+i{k}_{0}{\mu }_{{{{{{\rm{r}}}}}},{{{{{\rm{p}}}}}}}{A}_{{{{{{\rm{p}}}}}}}}{({a}_{1}+{a}_{2})-i{k}_{0}{\mu }_{{{{{{\rm{r}}}}}},{{{{{\rm{p}}}}}}}{A}_{{{{{{\rm{p}}}}}}}}$$

where *k*_0_ is the propagation constant of EM waves in vacuum and *μ*_r,p_ is the relative permeability of materials in both the ENZ channel and ports. “*i*” is the imaginary unit and an *e*^-*iωt*^ time convention is first assumed and suppressed. To enable EM waves to tunnel perfectly through the narrow channel, it is necessary that *a*_1_ ≈ *a*_2_ and that *k*_0_*μ*_r,p_*A*_p_ is as small as possible, which means that an unattainable tunneling channel with a width approaching zero is required. In practice applications, however, channels with finite width introduce the reflection and reduce the transmission efficiency^9,10^. Taking inspiration from the concept of ENZ metamaterials, we exhibit two types of photonic doping to accomplish impedance matching and reduce reflection.

**Fig. 1:**
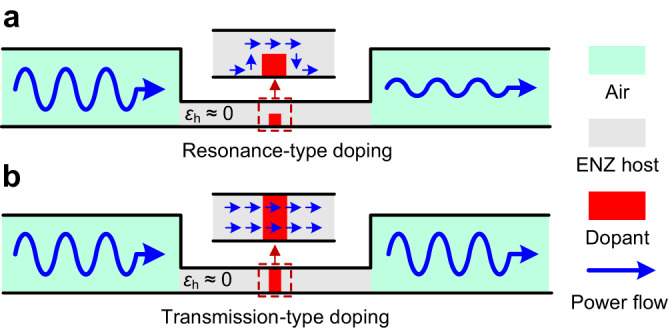
Supercoupling in ENZ metamaterials. Conceptual sketch of supercoupling that EM waves are squeezed and tunneled through an ENZ channel at the subwavelength scale. **a** Resonance-type doping and (**b**) Transmission-type doping are used for impedance matching to maximize the transmitted energy. The illustrations demonstrate the direction of Power flow, i.e., the Poynting vector near the dopants.

Figure 1 shows two types of photonic doping including the resonance-type and transmission-type ones. For the conventional resonance-type doping in Fig. 1a, we consider a rectangular dopant for easy processing and integration in applications. The dopant with high relative permittivity *ε*_d_ is located in the ENZ host, with its bottom edge in contact with the perfect electrical conductor (PEC) boundary. A space is left between its top edge and the PEC boundary. According to image principle^45^, the dopant can be equivalent to a rectangular dielectric rod doped in a 2D ENZ host^24^. At the impedance matching frequency, i.e., EMNZ frequency, partial energy couples into the dopant and constructs a 2D high-Q resonance. This part of energy is inevitably dissipated due to dielectric losses in the dopant. Other transmitted EM energy waves wrap around the dielectric dopant and squeeze through the narrow space left, as indicated by blue arrows in the inset^17,18^. Influenced by two singular corner points in the rectangular dopant, the EM waves that pass through the left space are strongly restricted, exhibiting a relationship between the width of the left space and transmission amplitude (see Supplementary Note 1). Trapped by both mechanisms, the efficiency of ENZ supercoupling assisted by resonance-type doping has no significant improvement compared to the general case^24^.

In contrast to the conventional resonance-type doping, the transmission-type doping in Fig. 1b, however, places the dopant in the middle of the ENZ channel with both top and bottom edges in contact with the PEC boundaries. At the frequency of impedance matching, also known as EMNZ frequency, the length of dielectric dopant is approximately a multiple of half the equivalent wavelength. A 1D, cosine-distributed, and low-Q resonance is formed in the dopant. As a result, when EM waves undergo two reflections to reach the incident side of the dopant, they always remain in the same phase as the incident waves, thus enhancing the transmission amplitude. The EM power flow transmits through the ENZ channels without turning, also as indicated by blue arrows in the inset. Compared to conventional resonance-type doping, the transmission-type of photonic doping reduces the *Q* factor and intensity of resonance in the dopant, thereby decreasing the dielectric losses and accomplishing the high-efficiency ENZ supercoupling. This enhancement, based on the reflection and in-phase superposition, can be described by the approach based on transmission line theory, which is demonstrated in the next section.

### Theoretical approach and numerical simulation

**Fig. 2:**
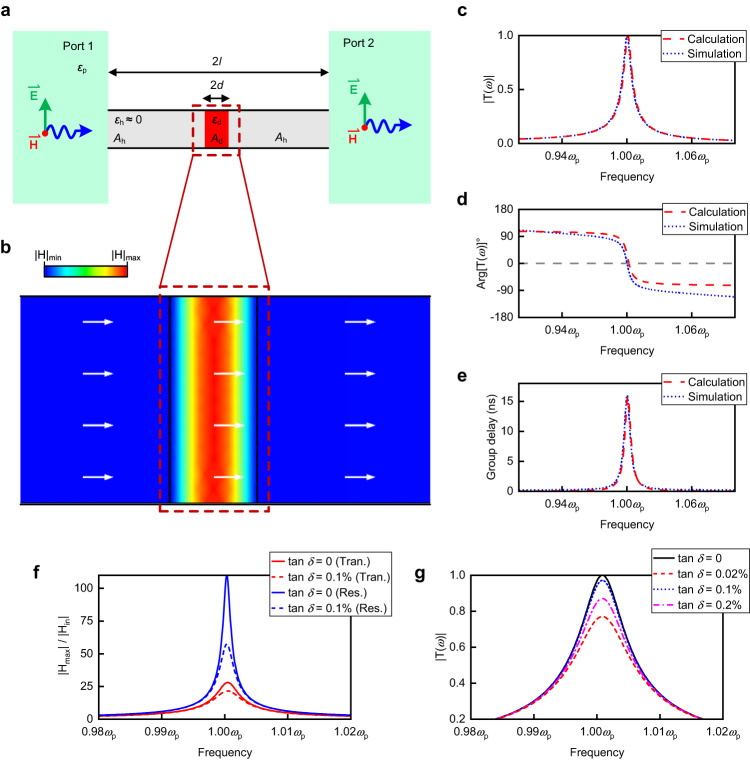
Theoretical approach and numerically simulated results of transmission-type doping. **a** The geometry of transmission-type doping. A dopant with the length 2*d* and relative permittivity *ε*_d_ is placed in the middle of the ENZ channel with the length 2 *l*. **b** Simulated results of magnetic field distribution in (**a**) at the EMNZ frequency. The white arrows reveal the direction of the EM energy fluids. **c** Calculated transmission amplitude, **d** phase (after calibration), and (**e**) group delay for the transmission-type doping in (**a**). Numerically simulated results are demonstrated for comparison. **f** Magnetic field enhanced coefficient |*H*_max_|/|*H*_in_| in both resonance-type and transmission-type doping. Results with different dielectric loss tangent tan *δ* are used for comparison. Trans., transmission-type doping. Res., resonance-type doping. **g** Calculated results of transmission amplitude for the dopants with different dielectric loss tangents in (**a**).

We demonstrate the geometry of the transmission-type doped ENZ channel in Fig. 2a. A dopant with a length of 2*d* and a relative permittivity of *ε*_d_ is located in the middle of the ENZ host with a length of 2*l* and a relative permittivity *ε*_h_ ≈ 0. The top and bottom edges of the dopant are in contact with the PEC boundaries. We assume that both sides of the channel are connected with parallel-plate waveguides filled with a medium of relative permittivity *ε*_p_, omitting an assumption that all materials have a relative permeability of *μ*_r_ = 1. That is to say, the waveguides for transmitting and receiving EM waves are with identical impedance for high transmission rate, i.e., impedance matching. Due to the different boundary conditions for the electrical fields, we cannot explain this doping approach with the existing theory of photonic doping^24,28^ (Detailed information can be found in Supplementary Note 2). Here, we analyze the supercoupling effect assisted by the transmission-type photonic doping based on the transmission line theory^36^. This approach facilitates the accurate calculation of the transmission response of the doped ENZ medium and allows for quantitative analysis of material losses.

Based on the transmission line theory, a segment of waveguide with the length *l* and relative permittivity *ε*_r_ can be equivalent to a transmission line with a parameter matrix. We consider both segments of the ENZ channel and the dopant as transmission lines, thus we can calculate their transfer matrices as

2 $${A}_{{{{{{\rm{d}}}}}}}=\left[\begin{array}{cc}\cos \left(\sqrt{{\varepsilon }_{{{{{{\rm{d}}}}}}}}\frac{2\omega d}{c}\right) & -\frac{i{\eta }_{0}}{\sqrt{{\varepsilon }_{{{{{{\rm{d}}}}}}}}}\,\sin \left(\sqrt{{\varepsilon }_{{{{{{\rm{d}}}}}}}}\frac{2\omega d}{c}\right)\\ -\frac{i\sqrt{{\varepsilon }_{{{{{{\rm{d}}}}}}}}}{{\eta }_{0}}\,\sin \left(\sqrt{{\varepsilon }_{{{{{{\rm{d}}}}}}}}\frac{2\omega d}{c}\right) & \cos \left(\sqrt{{\varepsilon }_{{{{{{\rm{d}}}}}}}}\frac{2\omega d}{c}\right)\end{array}\right]$$

3 $${A}_{{{{{{\rm{h}}}}}}}=\left[\begin{array}{cc}\cos \left(\sqrt{{\varepsilon }_{{{{{{\rm{h}}}}}}}}\frac{\omega (l-d)}{c}\right) & -\frac{i{\eta }_{0}}{\sqrt{{\varepsilon }_{{{{{{\rm{h}}}}}}}}}\,\sin \left(\sqrt{{\varepsilon }_{{{{{{\rm{h}}}}}}}}\frac{\omega (l-d)}{c}\right)\\ -\frac{i\sqrt{{\varepsilon }_{{{{{{\rm{h}}}}}}}}}{{\eta }_{0}}\,\sin \left(\sqrt{{\varepsilon }_{{{{{{\rm{h}}}}}}}}\frac{\omega (l-d)}{c}\right) & \cos \left(\sqrt{{\varepsilon }_{{{{{{\rm{h}}}}}}}}\frac{\omega (l-d)}{c}\right)\end{array}\right]$$

where *ω* = 2*πf* is the angular frequency, *η*_0_ = 337 Ω is the vacuum wave impedance and *c* = 3 × 10^8^m/s is the speed of light in vacuum. After being connected with the feeding two ports, the total transfer matrix of the system is given by

4 $$A=\left[\begin{array}{cc}{({\eta }_{0}/\sqrt{{\varepsilon }_{{{{{{\rm{p}}}}}}}})}^{-\frac{1}{2}} & 0\\ 0 & {({\eta }_{0}/\sqrt{{\varepsilon }_{{{{{{\rm{p}}}}}}}})}^{\frac{1}{2}}\end{array}\right]{A}_{{{{{{\rm{h}}}}}}}{A}_{{{{{{\rm{d}}}}}}}{A}_{{{{{{\rm{h}}}}}}}\left[\begin{array}{cc}{({\eta }_{0}/\sqrt{{\varepsilon }_{{{{{{\rm{p}}}}}}}})}^{\frac{1}{2}} & 0\\ 0 & {({\eta }_{0}/\sqrt{{\varepsilon }_{{{{{{\rm{p}}}}}}}})}^{-\frac{1}{2}}\end{array}\right]$$

Through the transformation from transfer matrix to scattering matrix, the transmission coefficient can be written as

5 $${{{{{\rm{T}}}}}}(\omega )=\frac{2}{{A}_{11}+{A}_{12}+{A}_{21}+{A}_{22}}$$

where *A*_11_, *A*_12_, *A*_21_, and *A*_22_ are the four elements of the matrix *A*, and so forth. By selecting *ε*_d_ = 37, *d* = 0.42 cm, *l* = 7.5 cm, we calculate the transmission coefficient near *ω*_p_ = 2*π* × 3 × 10^9 ^rad/s and plot its amplitude, phase, and group delay responses in Fig. 2c–e, together with the numerically simulated results. After excluding the additional phase shift introduced by the ports and the dopant (detailed information can be found in Supplementary Note 3), all simulated results demonstrate a high agreement with calculations, illustrating the accuracy of our approach. Similar to the resonance-type doping approach^24^, the system simultaneously reaches the maximum amplitude, zero-phase shift, and maximum group delay near *ω*_p_, which are the typical features of EMNZ states. At this frequency, ENZ supercoupling occurs.

Despite the intrinsic difficulty of expressing *T*(*ω*) in a single simple equation, we can still perform some proximate analysis (detailed information is also demonstrated in Supplementary Note 3). From the proximate results, the length of the dopant approximately satisfies the following relationship

6 $$2d=\frac{m\pi c}{\omega \sqrt{{\varepsilon }_{{{{{{\rm{d}}}}}}}}}=\frac{m{\lambda }_{{{{{{\rm{d}}}}}}}}{2},\, m=1,2,3,{{{{\mathrm{..}}}}}.$$

Where *λ*_d_ is the equivalent wavelength in the dopant. That is to say, the length of the dopant corresponds to an integer multiple of half-wavelength with the factor *m*. We notice that the structural parameter *d* = 0.42 cm corresponds exactly to the case where *m* = 1 in Eq. (6). Here we plot the simulated magnetic distribution out-of-plane in Fig. 2b, which is uniform in the width direction and exact cosine-symmetric distribution of half a wavelength in the length direction. The EM power flow, i.e., the Poynting vector is also shown in the figure.

Figure 2b clearly shows the formation of a 1D resonance in the dopant. To provide a quantitative comparison of both the resonance intensity and losses with the 2D resonance supported by the resonance-type doping, we conduct simulations of the magnetic field enhanced coefficient |*H*_max_|/|*H*_in_| in both types of doping. The results are shown in Fig. 2f. Here, |*H*_max_| represents the maximum of magnetic field in the dopants, and |*H*_in_| represents the intensity of input magnetic field. As we can see, the enhanced coefficient for resonance-type doping is almost three times larger than that for transmission-type doping. It is well known the enhanced coefficient is proportional to the *Q* factor^45^. From a physical standpoint, we can understand the relationship between the enhanced coefficient, *Q* factor, EM energy, and losses in ENZ supercoupling. When the coefficient and *Q* factor are larger, the energy is more concentrated in the dopants instead of the ENZ channels. At this point, the same media losses result in a larger efficiency degradation for the more intense 2D resonance supported by resonance-type doping. As shown in Fig. 2f, the resonance-type doping exhibits a significant decrease in efficiency at the same loss tangent, which is consistent with the previous discussion. To achieve high-efficiency transmission, it is crucial to reduce resonance, as we have done in transmission-type doping. In this manner, we minimize losses and accomplish high-efficiency ENZ supercoupling assisted by the proposed transmission-type doping.

In the practical circumstances, microwave materials inevitably suffer from intrinsic losses, which are generally described by the imaginary parts of relative permittivity in materials^46^. Here we discuss the influence of lossy dopants as a complement for practical applications. Substituting the lossy permittivity *ε*_d_ = 37 (1+ i×tan *δ*), we calculated the transmission amplitude using the approach and plot it in Fig. 2e. Simulated results can be found in Supplementary Fig. 4, demonstrating high compliance between the calculated and simulated results. We can observe that as the losses increase, the transmission amplitude maintains at a level where it can be applied, although it decreases gradually. For low-loss dielectric ceramics with tan *δ* = 0.0002, the transmission efficiency exceeds 95% and hardly affects applications. For general ceramics with tan *δ* = 0.001, the transmission efficiency remains above 80%. These results demonstrate the potential for constructing high-efficiency supercoupling through transmission-type doping in practical applications.

### Supercoupling features in transmission-type doping approach

**Fig. 3:**
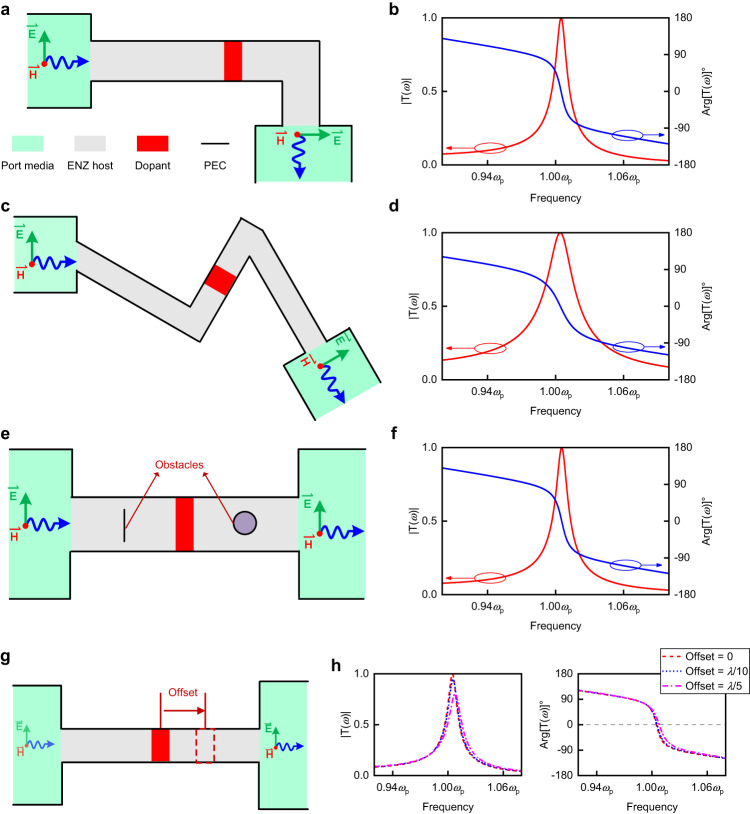
Numerical simulation results of supercoupling features in transmission-type doping approach. **a** The geometry of supercoupling in an asymmetric waveguide assisted by transmission-type doping. **c** The geometry of supercoupling in an irregular waveguide assisted by transmission-type doping. **e** The geometry of supercoupling in a waveguide with obstacles assisted by transmission-type doping. **b**, **d**, **f** Simulated results of both transmission amplitude and phase (after calibration) in (**a**), (**c**), and (**e**). Additional results of group delay and magnetic field distribution at the EMNZ frequency can be found in Supplementary Fig. 5. **g** The geometry of supercoupling assisted by transmission-type doping in waveguide with different dopant locations. The displacement of the dopant from the center of the waveguide is defined as the Offset. **h** Simulated results of both transmission amplitude and phase (after calibration) in (**g**).

While we have demonstrated the potential of constructing high-efficiency EM wave transmission assisted by transmission-type doping, we must carefully exclude the influence caused by other physical mechanisms, specifically the resonant coupling^25–27^. As a phenomenon that enhances transmission efficiency through the formation of Fabry-Perot transmission modes, resonant coupling requires a high degree of symmetry. This symmetry encompasses both the structural symmetry of the transmission waveguides and the positioning of the dopants at the center of the waveguides. Furthermore, resonant coupling occurs in various background media and cannot replicate the exceptional performance of ENZ supercoupling in sharp bends, corners, and obstacles. This limitation reduces the flexibility of the waveguide, which is not conducive to practical applications. To eliminate the possibility of resonant coupling and demonstrate the exhibition of supercoupling in our approach, we conduct simulation results in specific scenarios. In these cases, ENZ supercoupling enables full energy transmission with zero-phase shift, while resonant coupling is not available. The results are presented in Fig. 3.

In Fig. 3a, EM waves propagate in an asymmetric waveguide with a sharp bend. In Fig. 3c, an irregular waveguide is assumed, similar to the original studies on ENZ supercoupling. In Fig. 3e, two obstacles with PEC boundaries are placed in the waveguide. The length of the dopant in all systems is selected as 2*d* = 0.84 cm, corresponding to the EMNZ frequency near *ω*_p_ = 2*π* × 3 × 10^9 ^rad/s, which is the same as the model in Fig. 2. Original studies^8,17,19^ have demonstrated that supercoupling achieves full transmission with zero-phase advance in the above scenarios (for infinitely narrow ENZ channels), a feat not achievable by resonant coupling, apparently. From the simulated results in Figs. 3b, d, f, we can observe that all systems reach near total energy transmission with zero-phase shift at the EMNZ frequency. Additionally, as predicted, the magnetic field distribution at the EMNZ frequency (detailed results can be found in Supplementary Fig. 5) demonstrates a half-wavelength mode in the dopants. We also verify the stability of high-efficiency supercoupling assisted by transmission-type doping with different relative position of the ports (detailed information can be found in Supplementary Fig. 6). These results provide evidence that we establish high-efficiency supercoupling instead of resonant coupling. Assisted by transmission-type doping, we expand the infinitely narrow channel in ENZ supercoupling to a finite length, which holds significant potential for various applications.

However, in contrast to the resonance-type doping, the transmission-type doping approach demonstrates a strong dependence on the dopant locations. We consider a scenario where the dopant is not located in the middle of the ENZ channel but is displaced from the center. The geometry and simulated results of the scenario are shown in Fig. 3g, h. It is obvious that as the dopant moves away from the center, the amplitude decreases simultaneously. We clarify that this phenomenon arises due to specific modes in transmission-type doping, and it is not a feature of resonant coupling. In previous studies, researchers have demonstrated that the magnetic field distribution in 2D resonance-type doping follows a cylindrical or quasi-cylindrical mode^24,28^. This mode is not limited by spatial location in ENZ media, thereby making resonance-type doping exhibit an exotic position-independence feature. However, due to the PEC boundaries, this mode is not supported in transmission-type doping approach (detailed information can be found in Supplementary Note 2). Instead, there is a cosine-symmetric mode in the transmission-type dopant. When the dopant is off-center, a portion of the sine component is excited and the fields on both sides are no longer symmetric. Consequently, the impedances of the ENZ channels on both sides of the dopant become mismatched, resulting in a decrease in amplitude at the EMNZ frequency. Based on Fig. 3h, within the tolerance allowed by the existing machining process, the transmission efficiency remains high despite the decrease caused by the offset of dopant. In summary, we theocratically demonstrate high-efficiency supercoupling assisted by transmission-type doping. In the next section, we experimentally verify this approach in a 3D configuration with the assistance of waveguide-emulated plasma.

### 3D configuration and experimental verification

**Fig. 4:**
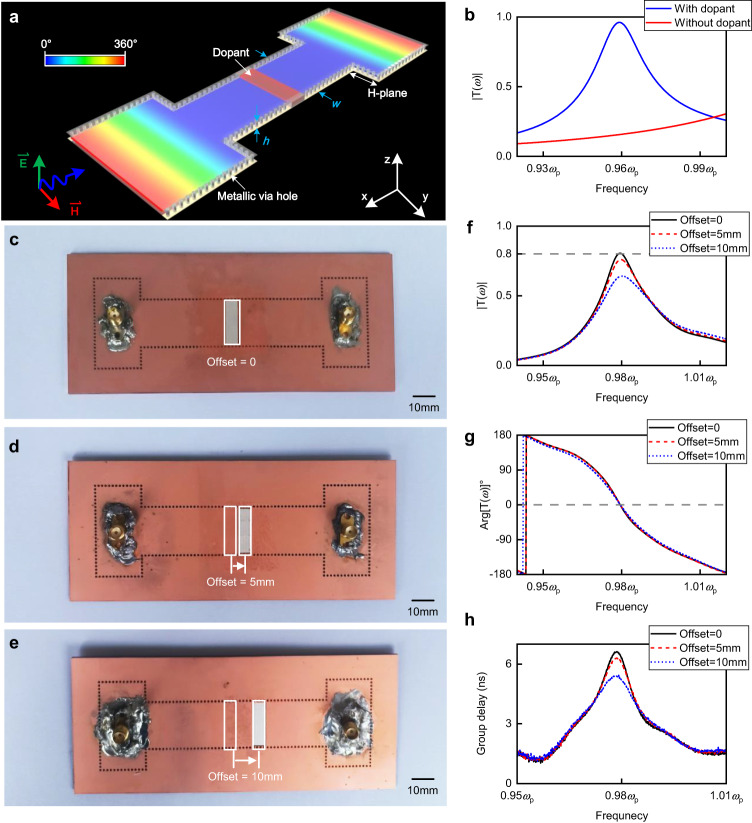
3D configuration and experimental verification of high-efficiency ENZ supercoupling. **a** The 3D configuration of a supercoupling waveguide assisted by transmission-type doping. Phase distribution (after calibration) of waves at the EMNZ frequency is schematically presented over the structure. **b** Simulated results for both configurations, with and without the dopant in (**a**). **c**–**e** Photographs of experimental setups with dopants located at different positions. The white cubes in the waveguides are dielectric ceramics used as dopants. **f** Experimental transmission amplitude (after calibration), (**g**) phase (after calibration), and **(h**) group delay in (**c**–**e**).

To extend the concept of ENZ metamaterials from the 2D ideal concept to practical applications, waveguide-emulated ENZ medium^37,38^ is used, which avoids the deteriorating effects of plasmonic losses. In this way, the TE_10_ mode of waveguides has a dispersive permittivity function *ε*_eff_(*ω*) = *ε*_f_ − *c*^2^*π*^2^ / *ω*^2^*w*^2^, where *w* is the width of the waveguide in the direction of the H-plane and *ε*_f_ is the relative permittivity of media in waveguides. This indicates the waveguide structure emulates the ENZ medium near its cut-off frequency. The 3D configuration of high-efficiency ENZ supercoupling is schematically demonstrated in Fig. 3a. For ease of manufacturing, we utilize the SIW microwave package technology to process the ENZ waveguide in a dielectric substrate^39,40^. Both top and bottom sides are covered by metals, which act as electrically conductive boundaries. Rows of metallic via holes are fabricated as metallic boundaries, benefiting from the SIW technique. Compared to the case without a dopant, a significant enhancement of transmission amplitude can be observed from the simulated spectra in Fig. 4b, indicating the occurrence of high-efficiency ENZ supercoupling. Based on this configuration, we experimentally verify the approach of high-efficiency ENZ supercoupling, while also considering the position-dependence of the dopant.

The photographs of three experimental setups with different dopant locations are displayed in Fig. 4c–e, respectively. All devices have a thickness of 1.01 mm and are made of dielectric materials with a relative permittivity of *ε*_r_ = 2.2, and a dielectric loss tangent of tan *δ*_ε_ = 0.0009. Two pieces of copper, covering the top and bottom, which act as PEC boundaries during the measurement, are omitted in the photographs. Rows of metallic via holes are punched in the devices, constructing a shape like a letter “H” with a narrow ENZ channel that has a width of 20 mm, corresponding to the cutoff frequency of *ω*_p_ = 2*π* × 5.065 × 10^9 ^rad/s. The length of the channel is 78 mm. Dielectric ceramic blocks are precisely embedded in the non-metallic via hole left in experiment setups. All dielectric blocks have a length of 5.4 mm and are made of a material with a relative permittivity *ε*_d_ = 37 and a dielectric loss tangent tan *δ* = 0.0002. The entire structures are suitable for large-scale integrated processing using the existing printed-circuit-board technology.

The measurements of transmission amplitude, phase, and group delay of the experimental setups are shown Fig. 4f–h. Combining these figures, we can see that the transmission and group delay reach maxima with zero-phase shift at 0.98*ω*_p_, which confirms our prediction of the EMNZ point. For the configuration without dopant offset, the maximum transmission efficiency is greater than 80%, and the maximum group delay is greater than 6 ns. The experimental results are in good agreement with the simulated results, while the additional losses observed may be attributed to the materials and machining accuracy (simulated results of 3D experimental configurations can be found in Supplementary Fig. 8). Compared to the original works, two experiments without photonic doping for general ENZ supercoupling exhibit maximum transmission amplitudes of less than −6 dB (50%)^10^ and approximately −5 dB (56%)^9^; whereas a resonance-type doping SIW shows a maximum transmission amplitude of approximately 60%^24^. In other words, the configuration accomplishes an efficiency enhancement of at least 20%, despite the fact that the propagation of EM waves through dielectric substates introduces more losses than through air. This illustrates that transmission-type doping exhibits higher efficiency and lower loss compared to resonance-type doping in experiments simultaneously. Additionally, as predicted, the transmission-type approach demonstrates a strong dependence on the dopant locations. With an increase in the offset, both the transmission amplitude and group delay decrease. However, in the case where offset = 10 mm, the transmission amplitude still remains more than 60%, which is higher than that in the original works. These experimental results demonstrate that we successfully realize the high-efficiency ENZ supercoupling assisted by the transmission-type photonic doping.

**Fig. 5:**
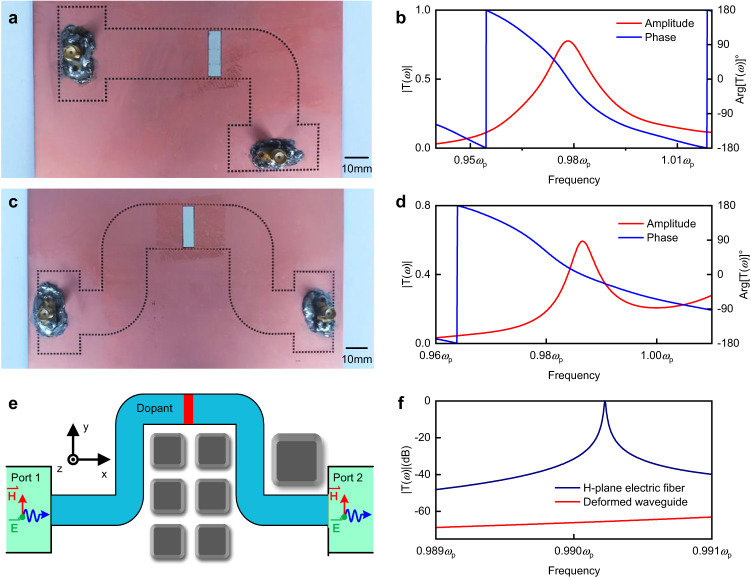
Experimental verification of supercoupling features and flexible H-plane electric fiber. **a** The photograph of an experimental setup for supercoupling in an asymmetric waveguide assisted by transmission-type doping. **b** Experimental results of both transmission amplitude and phase (after calibration) in **(a). c** The photograph of an experimental setup for supercoupling in a bending waveguide assisted by transmission-type doping. **d** Experimental results of both transmission amplitude and phase (after calibration) in (**c**)**. e** An example application for *H*-plane electric fiber. An arbitrary-shaped waveguide is deployed through the obstacles to propagate the EM waves in a complex environment. **f** Simulated transmission amplitude in dB in (**e**).

For potential applications of the transmission-type doping approach, we also experimentally verify the supercoupling features in 3D configurations, including an asymmetric waveguide and a bending waveguide. The photograph of the asymmetric waveguide is displayed in Fig. 5a. To ensure that the effective relative permittivity remains constant, as dictated by the dispersive equation *ε*_eff_(*ω*) = *ε*_f_ − *c*^2^*π*^2^ / *ω*^2^*w*^2^, and is near-zero around the cut-off frequency, we implement a quarter-circle structure at the corner. Apart from replacing the total length of the waveguide with 100 mm, all other parameters are kept the same as experimental setups in Fig. 4. The experimental results in Fig. 5b demonstrate a maximum transmission amplitude around 75% with zero-phase shift at 0.98*ω*_p_ for the asymmetric waveguide. As for the bending waveguide, whose photograph is displayed in Fig. 5c, the total length is 180 mm. Though longer ENZ channel introduces additional losses, the maximum of transmission amplitude still remains above 60%, as depicted in Fig. 5d. For comparison, the maximum of experimental transmission amplitude in a bending waveguide assisted by resonance-type doping is 50%^24^. These experimental results are consistent with the predictions of the supercoupling properties discussed in Fig. 3 and also serve as evidence for high-efficiency ENZ supercoupling. High-efficiency supercoupling holds great potential for high-efficiency EM wave propagation, which can be instrumental in future integrated circuit innovations. Here, we provide an example of the “H-plane electric fiber” to illustrate this potential.

The concept and configuration of H-plane electric fibers are shown in Fig. 5e. In a complex scenario, various obstacles, represented by blocks in the figure, prevent the possibility of propagation using straight waveguides. In the absence of dopants, the propagation of EM waves in the deformed waveguide will introduce more than 60 dB of loss, as shown in Fig. 5f. With the assistance by transmission-type doping, however, the waveguide avoids the losses caused by complicated bending, enabling a high-efficiency transmission of EM waves in such harsh conditions. This configuration overcomes the significant discontinuity in the H-plane, much like the flexibility of light fiber, hence the name “H-plane electric fibers”. The key distinction is that these electric fibers allow high-efficiency transmission of electromagnetic waves at sub-wavelength scales.

Compared to the E-plane electric fibers, which overcome the discontinuity in E-plane and are assisted by resonance-type doping^24^, H-plane electric fibers have three irreplaceable advantages. Firstly, for practical applications with lossy dopants, experimental results demonstrate the H-plane electric fibers have at least 10% efficiency enhancement in the presence of losses in the propagated media. This value is expected to increase further when dielectric substrate losses are resolved. Secondly, the profile height of E-plane electric fibers is limited to greater than *h* > *λ*_d_/4, where *λ*_d_ represents the effective wavelength in the dopant (detailed information can be found in Supplementary Note 1). For example, at *ω*_p_ = 2 *π* × 5.065 × 10^9 ^rad/s, the minimum height of E-plane electric fibers is 2.5 mm, which restrict their applications. However, H-plane electric fibers overcome this limitation, as evidenced by our experimental setup with a plate thickness of only 1 mm. This allows the placement of H-plane electric fibers in ultrathin scenarios. Thirdly, H-plane electric fibers eliminate height-wise skipping of E-plane electric fibers, thus ensuring a uniform thickness throughout the entire configurations. This provides convenience for manufacturing through a variety of processes in microwave packages, as illustrated in our experimental setups for an example of SIW.

As we all know, conventional lumped components are typically integrated with microstrip-type structures, which are currently the most commonly used architectures in microwave applications. With the frequency increasing, the capability of microstrip-type structures to confine EM energy decreases significantly, manifested as the increasing radiation loss and crosstalk^41,42^. In contrast, waveguides can prevent energy leakage during propagation, potentially providing competitive and favorable solutions for the next-generation integrated circuit architectures. In this direction, many lumped components have already been investigated^47–49^. The H-plane electric fibers, based on high-efficiency ENZ supercoupling, provide a potential alternative for low-loss conduits in waveguide-integrated structures, which is of great significance for the development of microwave-integrated devices. We confirm that our work will make a dramatic revolution in the area of microwave engineering.

## Discussion

In this work, we demonstrate the high-efficiency ENZ supercoupling based on our proposed transmission-type doping. This type of doping replaces high-Q 2D resonant modes in the dopant with low-Q 1D modes, thereby reducing dielectric losses in the photonic doping. Assisted by the transmission-type doping, we achieve high-efficiency ENZ supercoupling. On the basis of transmission line theory, we formulate an approach of transmission-type doping that agrees with the simulated results and provides a clear physical understanding of the reduction in losses. Moreover, we showcase supercoupling features in our approach through simulation, including achieving full transmission with zero-phase advance through distorted waveguides with bends, corners, and obstacles. Benefiting from the waveguide-emulated ENZ medium, we theoretically propose and experimentally verify 3D configurations based on high-efficiency ENZ supercoupling, considering both dopant position dependence and arbitrary-shaped waveguides. Experimental results show at least 20% enhancement compared to the original photonic doping design^9,10,24^, thereby achieving the highest ENZ supercoupling efficiency. Based on the high-efficiency ENZ supercoupling, we propose the H-plane electric fibers, which enable the propagation of EM waves in waveguides under a wide range of complicated conditions. The H-plane electric fibers demonstrate irreplaceable advantages, including high efficiency, low profile height and ease of integration through microwave packages. From the application example of flexible arbitrary-shaped H-plane electric fiber, we confirm that our work has significant implications for efficiency enhancement for the geometry-irrelevant circuit devices based on index-near-zero metamaterials.

## Methods

### Full-wave simulation

Simulated results for the transmission coefficients, the enhanced coefficient and the field distribution of 2D structures with ideal ENZ media (Fig. 2 and Fig. 3) are performed by using the RF module of the finite-element-method (FEM) commercial software COMSOL Multiphysics® v5.0 (available at www.comsol.com). The parameters of structures and materials are set as those in the main text. We use PEC as the boundaries, and two rectangular ports with TM modes as the excited sources. All the numerical simulations of 3D structures (Figs. 4b, 5f) are performed using the ANSYS HFSS®. The simulated models are the same as the concept schematics and photographs of devices. We also use PEC as the structure and boundaries. Except for the materials mentioned in the main text, all relative permittivity parameters are from the material library in the software. 50-Ohm lumped ports are used for excitations at the position where the SMA connectors are located.

### Experiment setup

The prototypes of transmission-type doped SIW presented in Figs. 4c–e, 5a, c are fabricated on a 1.01 mm thickness substrate made of Rogers RT/duroid 5880 (tm) using standard printed circuit board (PCB) techniques. The relative permittivity is *ε*_r_ = 2.20, and dielectric loss tangent is tan *δ* = 0.0009. The metallic and non-metallic via holes are made by hollowing out the predesigned geometries from the substrate, and copper is printed on the top and bottom surfaces of the substrate to serve as boundaries. After assembling the SIW and dopant, two copper skins are covered as the PEC boundaries. The SMA connector with its inner probe being 1.01 mm in length is inserted at a distance of approximately 10 mm away from the port’s end to excite the waveguide. The vector network analyzer Agilent N5227B, connected to the ports via 50 Ω coaxial lines, evaluates the transmission coefficients of the fabricated samples. Two 1.85 mm to 3.5 mm adapter connectors are used to connect with the ports, and a total insertion loss of 0.5 dB is calibrated in the experimental results.

### Supplementary information

Supplementary Information

## Data Availability

The simulation and experiment data that support the findings of this study are available from the corresponding author upon request.
