# Supplementary material for: Transmission-type photonic doping for high-efficiency epsilon-near-zero supercoupling
Source: Nat Commun. 2023 Oct 3;14:6154. doi: 10.1038/s41467-023-41965-5 (PMC10547686; doi:10.1038/s41467-023-41965-5)
Supplement: Supplementary file 1 — Supplementary Information [file 41467_2023_41965_MOESM1_ESM.pdf]

Transmission-type photonic doping for high-efficiency ENZ supercoupling

Yan. et al.

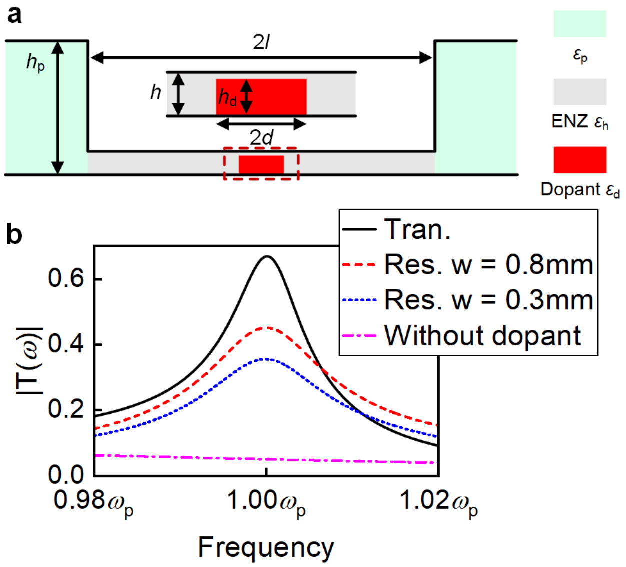

Supplementary Figure 1 | Simulated results in lossy ENZ media. (a) The geometry and parameter definitions of lossy ENZ supercoupling. **(b)** Simulated transmission amplitude of different types of doping in **(a)**. Tran., transmission-type doping. Res., resonance-type doping.

Supplementary Note 1. Space-constrained resonance-type doping

Unlike the ideal 2D photonic doping system with an infinite background, the ENZ host in supercoupling channels is a space-constrained system. As a result, resonance-type doping is limited in this system. In this section, we provide an explanation of this issue through both theoretical analysis and numerical simulation.

We assume a case as same as Fig. 1b in the main text, i.e., resonance-type doped ENZ channels. The parameters are set as follows: the length of the dopant is $2d$; the width of the dopant is h_d; the relative permittivity of the dopant is ε_d; and the area of the ENZ channel is A. From the formula of 2D traditional resonance-type doping[1,2], we get

$$\mu_{\mathrm{eff}}(\omega) \approx 1 + \frac{64*4dh_d}{\pi^4 A}\frac{\omega^2}{\omega_{\mathrm{pmc}}{}^2 - \omega^2},\ \omega_{\mathrm{pmc}} = \frac{c}{\sqrt{\varepsilon_d}}\sqrt{(\pi/2d)^2 + (\pi/2h_d)^2} \quad \text{(S1)}$$

Where c is the speed of light in vacuum. From Eq. (S1), we get

$$\left(\frac{\pi}{2d}\right)^2 = \frac{\omega_{\mathrm{pmc}}^2 \varepsilon_d}{c^2} - \left(\frac{\pi}{2h_d}\right)^2 \quad \text{(S2)}$$

Because of $(\pi/2d)^2 > 0$ for finite d, we can calculate the range of h as

$$h_{\mathrm{d}} > \frac{\lambda}{4\sqrt{\varepsilon_{\mathrm{d}}}} = \frac{\lambda_{\mathrm{d}}}{4} \tag{S3}$$

Where λ is the wavelength of the PMC frequency in a vacuum, and λ_{d} is the wavelength in the dopant. In other words, to accommodate a dopant with a height of h_{d}, we need an ENZ channel with a thickness h at least h_{d}. For a better understanding of this issue, researchers have demonstrated that the magnetic field distribution in 2D resonance-type doping follows a cylindrical or quasi-cylindrical mode, as shown in previous studies[1,2]. To accommodate this quasi-cylindrical mode in the resonance-type doping, the dopant needs to have a length of at least half a wavelength in both the horizontal and vertical directions. Furthermore, based on the image principle[3], the minimum height should be greater than λ_{d} / 4. With the given parameters (ε_{d} = 37 and $\omega_{\mathrm{p}} = 2\pi\times3\times10^9$ rad/s) in the main text, we have calculated that the minimum width of the ENZ channel is 4.11 mm, approaching the maximum thickness that can be achieved with the current SIW manufacturing technique. Resonance-type doping cannot meet the requirements of the ultranarrow thickness and profiles of substrates, which limits its practical application in the field of microwave-integrated devices.

Moreover, in the simulation of lossy ENZ media, the transmission efficiency of resonance-type doping demonstrates a strong dependence on the remaining space between the top of the dopant and the PEC boundary. This is mainly because of the two singular points in the rectangular dopant of resonance-type doping[2]. Here we analyze this dependence through simulation, whose geometry is shown in the Fig. S1a. We set the parameters as h_{p} = 30 mm, h = 6 mm, $2l$ = 80 mm, and $\varepsilon_{\mathrm{d}} = 37 + 2\times10^{-5}i$. We adjust the size of dopant flexibly to enable the EMNZ frequency $\omega_{\mathrm{p}} = 2\pi\times3\times10^9$ rad/s. To emulate the plasmonic implementation for the general ENZ media, we set the relative permittivity of ENZ host satisfies the Durde-model: $\varepsilon_{\mathrm{h}}(\omega) = 1 - \omega_{\mathrm{p}}^2/\omega(\omega+0.03i\omega_{\mathrm{p}})$, where $\omega = 2\pi f$ is the angular frequency in radians per second[4]. Simulated results are shown in Fig. S1b. As can be observe, the ranking of the transmission amplitude from high to low for several doping scenarios is as follows: transmission-type doping, resonance-type doping, and the undoped general case. In other words, our transmission-type doping is currently the most efficient way to achieve ENZ supercoupling, as demonstrated by the high-efficiency ENZ supercoupling presented in the main text. For resonance-type doping with different remaining width w, the transmission amplitudes exhibit different behaviors. A larger remaining width has better transmission efficiency. This is because a too narrow remaining width may cause unnecessary reflection. As discussed earlier, the remaining width is space-constrained in resonance-type

doping of SIW integration. Therefore, the resonance-type doping of ENZ supercoupling is unable to meet the requirement for long-distance transmission due to this space limitation.

The discussion in this section provides supplementary results for our transmission-type photonic doping approach in achieving high-efficiency ENZ supercoupling.

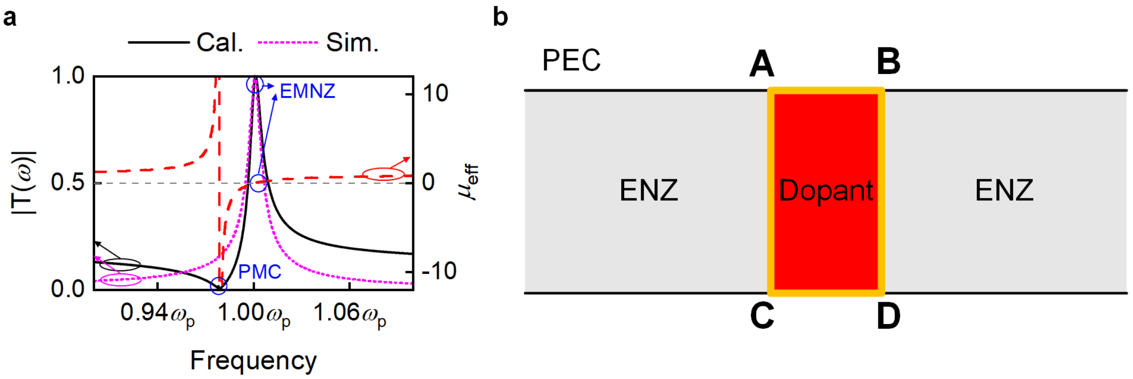

Supplementary Figure 2 | Conventional analysis of transmission-type photonic doping. (a) Calculated results for both effective relative permeability and transmission amplitude of transmission-type doping using traditional photonic doping theory. A simulated transmission amplitude is demonstrated for comparison. **(b)** Schematic for boundary conditions analysis for transmission-type doping.

Supplementary Note 2. Conventional analysis of transmission-type photonic doping

In this section, we attempt to analyze transmission-type doping using classical photonic doping theory to illustrate the limitations of conventional theory in addressing this issue[1]. We notice that the magnetic field distribution in the width direction in Fig. 2c of the main text is uniform, thus only the field in the length direction satisfies the passive scalar Helmholtz equation (SHE)

$$\frac{\partial^2}{\partial x^2} H_z(x) + k^2 H_z(x) = 0 \tag{S4}$$

where $k^2 = \omega^2\mu\varepsilon$. As the dielectric block is placed in the center, the magnetic field has an even symmetric distribution on both sides of the center. Therefore, there is only one SHE eigenvalue of the cosine function for the $H_z(x)$ component. According to the theory of conventional photonic doping, we obtain the normalized magnetic field $\psi_z(x)$ = $cos(kx)/cos(kd)$ in the dopant which satisfies the boundary conditions of the ENZ host. Here $k = \omega / c\sqrt{\varepsilon_d}$,where ε_d is the relative permittivity of the dielectric dopant. According to the general photonic doping formula equation, we get[2,5]

$$\mu_{eff} = 1 + \frac{1}{2l}\left(\frac{2\tan(kd)}{kd} - 2d\right) \tag{S5}$$

$$\mathrm{T}(\omega) = \frac{2}{2\cos(\sqrt{\varepsilon_h \mu_{eff}}\frac{\omega l}{c}) - i(\sqrt{\frac{\varepsilon_h}{\mu_{eff}\varepsilon_p}} + \sqrt{\frac{\mu_{eff}\varepsilon_p}{\varepsilon_h}})\sin(\sqrt{\varepsilon_p \mu_{eff}}\frac{\omega l}{c})} \tag{S6}$$

With the same variable definitions as those in the main text, we generate Fig. S2a to display the calculated results of effective permeability and transmission amplitude, along with the corresponding simulated result. We notice that the predicted PMC mode in the conventional theory is prohibited in the simulated result. We provide an explanation from the perspective of boundary condition analysis. Here we replot the dopant area of transmission-type doping in Fig. S2b and label the four vertices of the rectangular dopant with A, B, C, and D. At the PMC scenario, the tangent components of the magnetic field on boundary A-B-C-D are exactly zero. However, A-B and C-D are also in contact with the PEC boundaries, causing the tangent components of the electric field on A-B and C-D to also vanish. According to the Uniqueness Theorem[3], only the mode with zero electric fields or zero magnetic fields can be supported within the dopant. Because the mode is meaningless, the PEC effect cannot appear. The discussion in this section demonstrates that the conventional resonance-type doping theory cannot be used to describe the new approach of transmission-type doping.

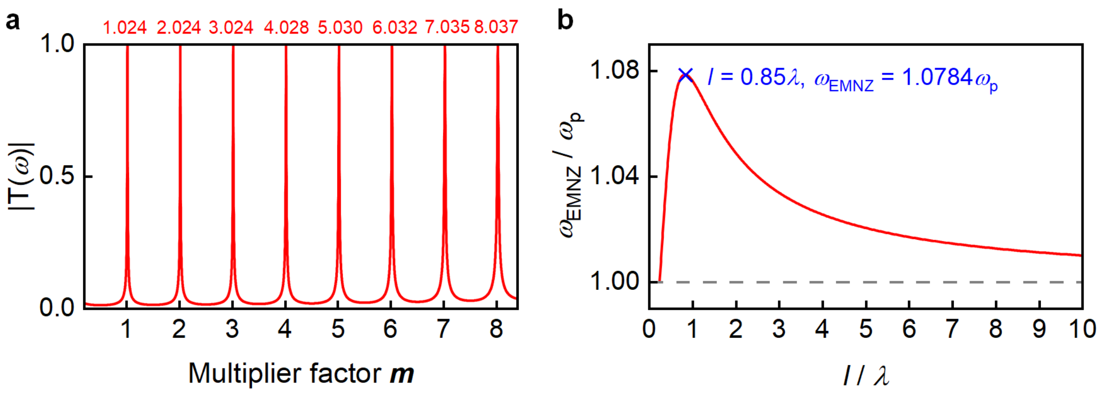

Supplementary Figure 3 | Numerical calculations of EMNZ frequency of transmission-type doping. (a) The transmission amplitude changes with the different lengths of dopants. Normalized lengths are the multiplier factor ***m*** of a half of the wavelength in the vacuum. The numbers above the figure mark the specific values of ***m*** for the maximum of transmission amplitude. **(b)** The normalized EMNZ frequency changes with the length of the ENZ channel *l*.

Supplementary Note 3. Proximate analysis of our new approach in the main text

We firstly begin our proximate analysis from the simplest case. For an ideal assumption, i.e., $\varepsilon_{\mathrm{h}} \equiv 0$ and $l \approx d$ in Eq. (3) in the main text, A_{h} degenerates to a second-order unit matrix. The dopant is in contact with the ports approximately directly, hardly ever passing through the ENZ channels at this time. The transmission coefficient can be written as[2,5]

$$\mathrm{T}(\omega)=\frac{2}{2\cos\left(\sqrt{\varepsilon_{\mathrm{d}}}\,\frac{2\omega d}{c}\right)-i\left(\sqrt{\frac{\varepsilon_{\mathrm{d}}}{\varepsilon_{\mathrm{p}}}}+\sqrt{\frac{\varepsilon_{\mathrm{p}}}{\varepsilon_{\mathrm{d}}}}\right)\sin\left(\sqrt{\varepsilon_{\mathrm{d}}}\,\frac{2\omega d}{c}\right)} \tag{S7}$$

From the transmission coefficient, we can get the amplitude and angle as

$$\left|\mathrm{T}(\omega)\right|=\frac{2}{\sqrt{4-\left(\sqrt{\frac{\varepsilon_{\mathrm{d}}}{\varepsilon_{\mathrm{p}}}}+\sqrt{\frac{\varepsilon_{\mathrm{p}}}{\varepsilon_{\mathrm{d}}}}-2\right)\sin^{2}\left(\sqrt{\varepsilon_{\mathrm{d}}}\,\frac{2\omega d}{c}\right)}} \tag{S8}$$

$$\mathrm{Arg}\left(\mathrm{T}(\omega)\right)=\arctan\left(\frac{\sqrt{\frac{\varepsilon_{\mathrm{d}}}{\varepsilon_{\mathrm{p}}}}+\sqrt{\frac{\varepsilon_{\mathrm{p}}}{\varepsilon_{\mathrm{d}}}}}{2}\tan\left(\sqrt{\varepsilon_{\mathrm{d}}}\,\frac{2\omega d}{c}\right)\right) \tag{S9}$$

According to the square inequality, $\sqrt{\varepsilon_\mathrm{d}/\varepsilon_\mathrm{p}}+\sqrt{\varepsilon_\mathrm{p}/\varepsilon_\mathrm{d}}>2$, here $|T(\omega)|$ get the maximum when $\sin(\sqrt{\varepsilon_\mathrm{d}}\,2\omega d/c)=0$, which the length $2d$ of the dopant satisfies Eq. (6) in the main text. The phase $\mathrm{Arg}(T(\omega)) = 0$ at this moment, heralding that we have got the EMNZ state.

For the general case, we accurately determine the difference between our approximation and the actual situations through numerical calculations. Our findings reveal that our approximations are highly accurate. Here we prove this through two calculated results which has shown in Fig. S3. In Fig. S3a, we calculate the transmission amplitude at different dopant lengths to determine the dopant lengths corresponding to the occurrence of the EMNZ states. By normalizing the length d using the Eq. (6) in the main text, we obtain a series of multiplier factors $\boldsymbol{m}$ which corresponding to the EMNZ states. The specific values are marked at above the figure. These m values are very close to the integer values as our theoretical prediction, indicating that the approximation is feasible under the simulation conditions. In order to analyze the shift of the EMNZ frequency due to this approximation, we calculate the normalized EMNZ frequency changing with the length of ENZ channel $2l$. As we can see, with the gradual increasing in the length channel length $2l$, the EMNZ frequency first undergoes a blue shift and then a red shift. The maximum frequency shift occurs at $l = 0.85\lambda$, with a shift of $0.08\omega_\mathrm{p}$. This error is completely acceptable in engineering terms. In other words, integer multiples of half-wavelength modes are formed within the dopant at EMNZ frequencies. Since the dopant serves as a crucial path for EM waves in ENZ supercoupling, it introduces an additional phase shift of 180° or 360°. In the main text, we have calibrated this phase to ensure consistency with concepts presented in previous work[1,2]. In a short summary, in this section, we show the basis for the approximate estimate of Eq. (6) in the main text and illustrate the accuracy of our approximate estimate through precise calculations.

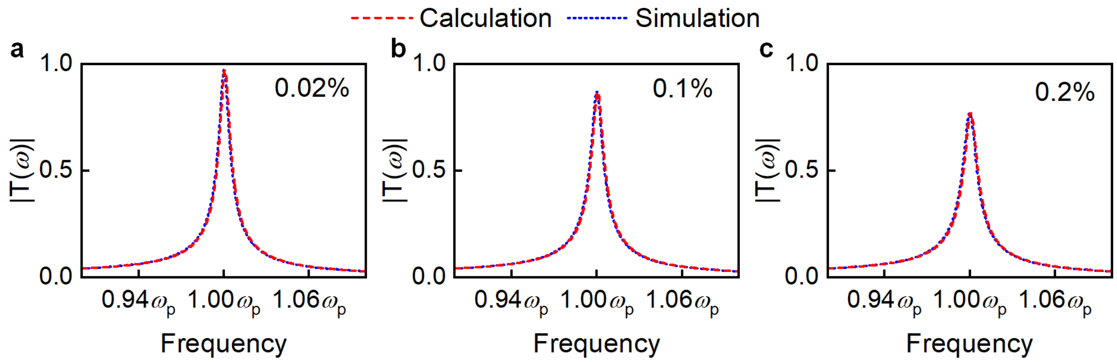

Supplementary Figure 4 | Calculated and simulated results for lossy dopant. Comparison between calculated and simulated results with different dielectric loss tangent for **(a)** 0.02%, **(b)** 0.1% and **(c)** 0.2%. The calculated results demonstrate high compliance with the simulated results, illustrating high accuracy for our transmission-type doping theory.

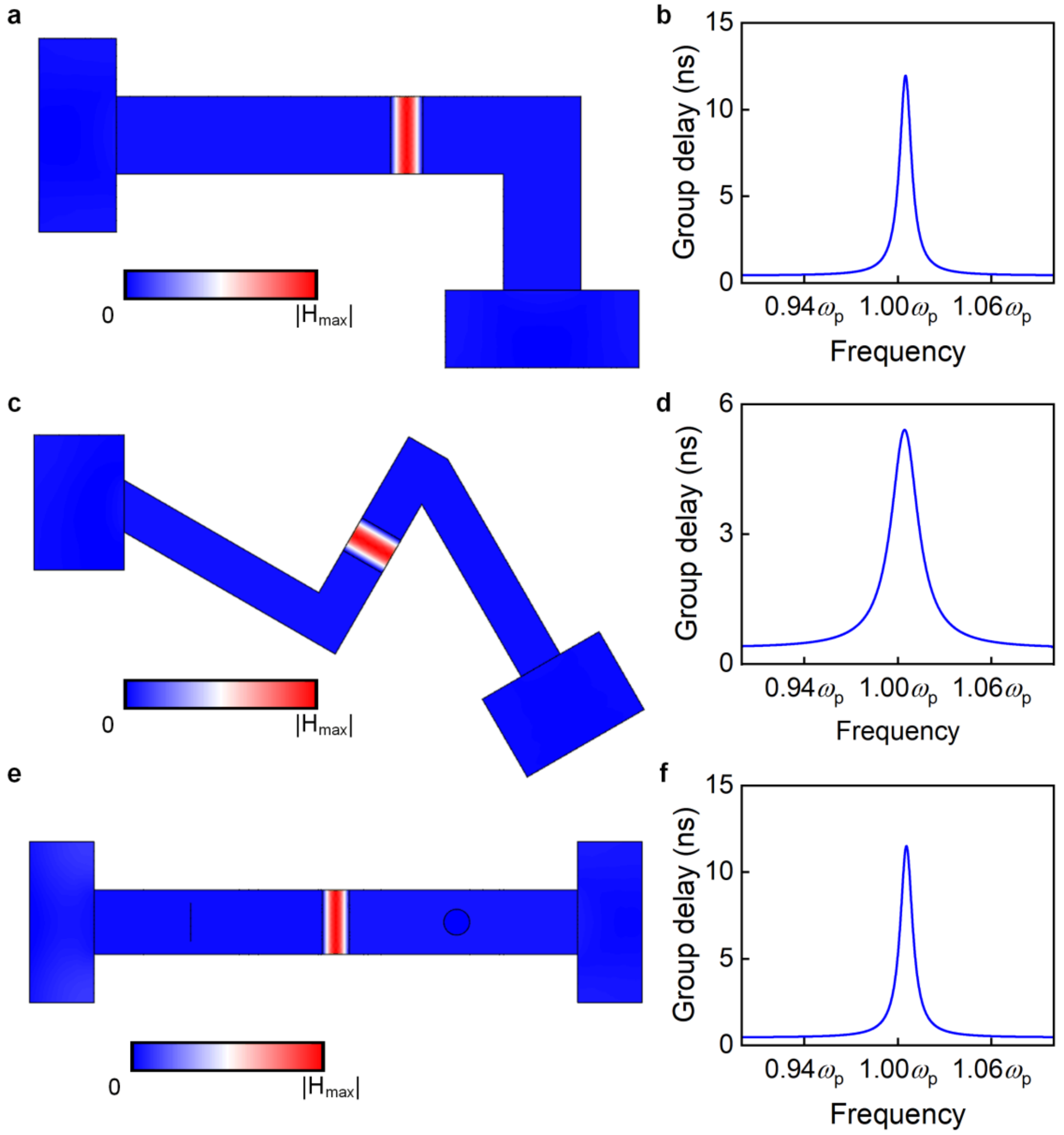

Supplementary Figure 5 | Additional numerical simulation results of supercoupling features in transmission-type doping approach. The magnetic field distribution of supercoupling in **(a)** an asymmetric waveguide, **(c)** an irregular waveguide, and **(e)** a waveguide with obstacles at EMNZ frequency assisted by transmission-type doping. The group delay in **(b)** an asymmetric waveguide, **(d)** an irregular waveguide, and **(f)** a waveguide with obstacles at EMNZ frequency assisted by transmission-type doping. This figure presents additional simulation results of supercoupling features for Fig. 3 in the main text.

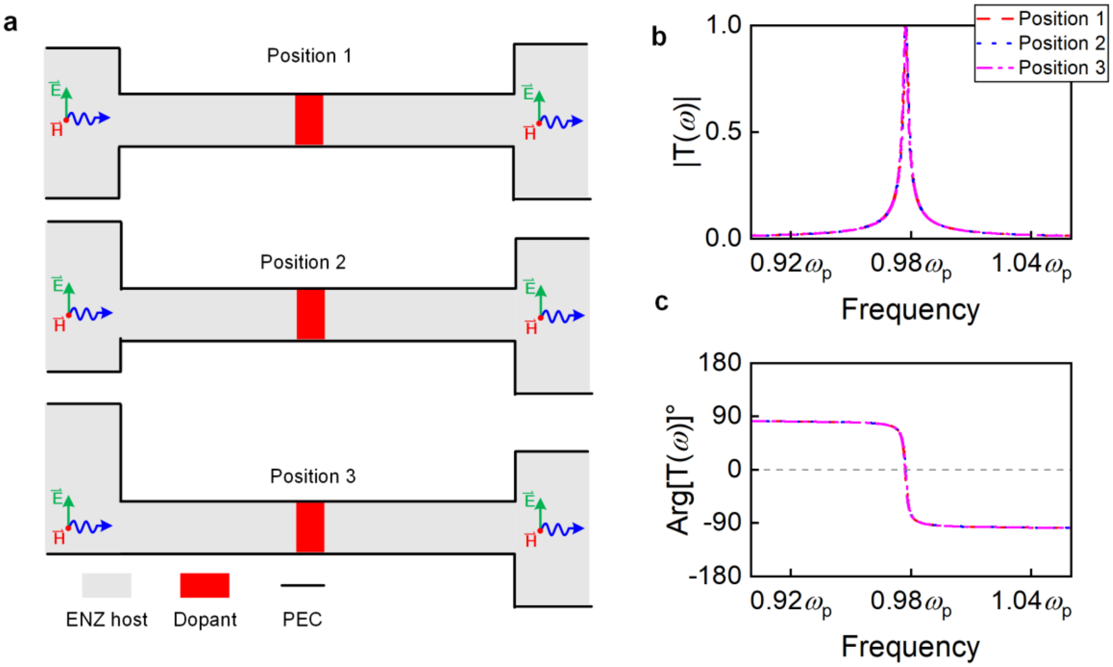

Supplementary Figure 6 | Numerical simulation results of supercoupling in waveguides with different relative position of the ports. (a) The geometries of supercoupling waveguides with different relative position of the ports. **(b)** Simulated transmission amplitude and **(c)** phase (after calibration) in **(a)**. As we can observe, the simulated transmission amplitude and phase for different relative positions of the ports maintain a high degree of stability, providing additional evidence of ENZ supercoupling assisted by transmission-type doping.

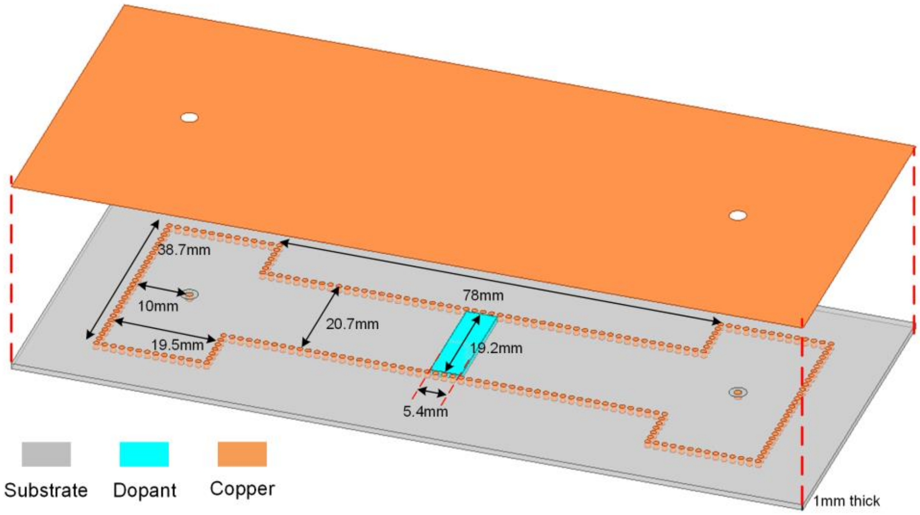

Supplementary Figure 7 | Geometry of supercoupling in a straight waveguide with offset = 0. 3D view of the supercoupling in a straight waveguide with offset = 0, i.e., the experimental setup in Fig. 4b in the main text. Parameter values are indicated in the Figure, and the parameters in the main text that slightly differ from the Figure are the results after the SIW equivalent. Metallic vias with radius 1 mm is arranged with the separation of 1.5 mm. The substrate is made in a dielectric material with $\varepsilon_r = 2.2$, while the dopant is made in a dielectric ceramic with $\varepsilon_r = 37$. The geometries for the cases offset = 5 mm and offset = 10 mm continue in the same way.

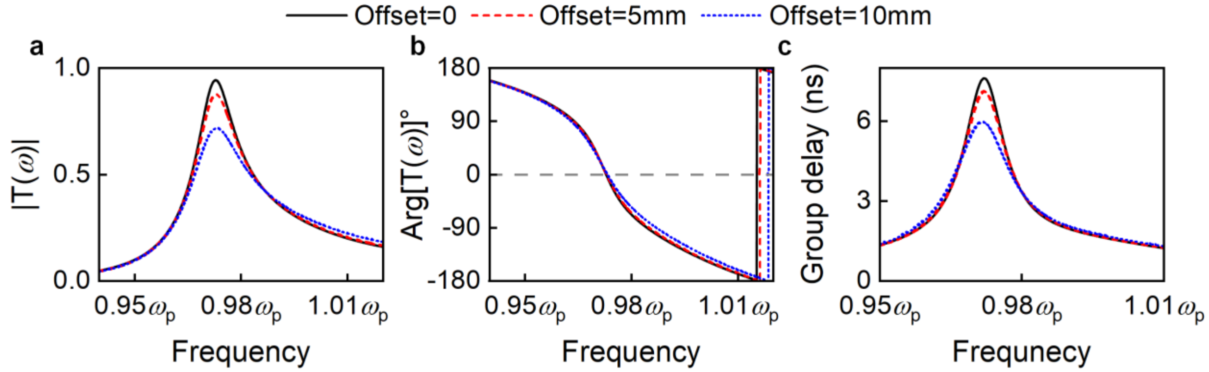

Supplementary Figure 8 | Simulated results of 3D configurations for straight experiment setups with different dopant locations. The 3D simulated results for experimental setups with different dopant locations in Fig. 4. **(a)** Transmission amplitude, **(b)** phase (after calibration), and **(c)** group delay. As we can observe, the experimental results in Fig. 4 are in good agreement with the simulated results. The slight additional loss and frequency drift are caused by the materials and processes used in the fabrication.

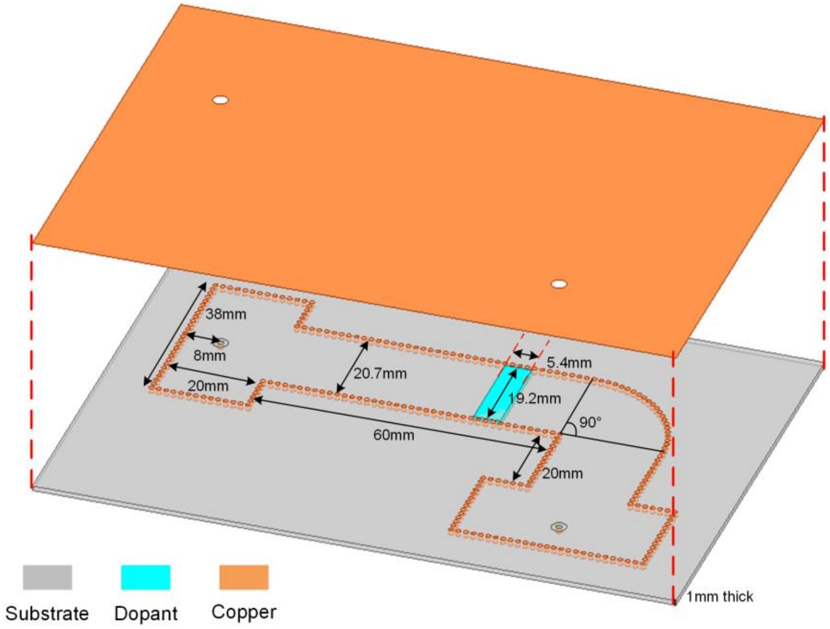

Supplementary Figure 9 | Geometry of supercoupling in an asymmetric waveguide. 3D view of the supercoupling in an asymmetric waveguide, i.e., experimental setup in Fig. 5a in the main text. Parameter values are indicated in the Figure, and the parameters in the main text that slightly differ from the Figure are the results after the SIW equivalent. Metallic vias with radius 1 mm is arranged with the separation of 1.5 mm. The substrate is made in a dielectric material with $\varepsilon_r = 2.2$, while the dopant is made in a dielectric ceramic with $\varepsilon_r = 37$.

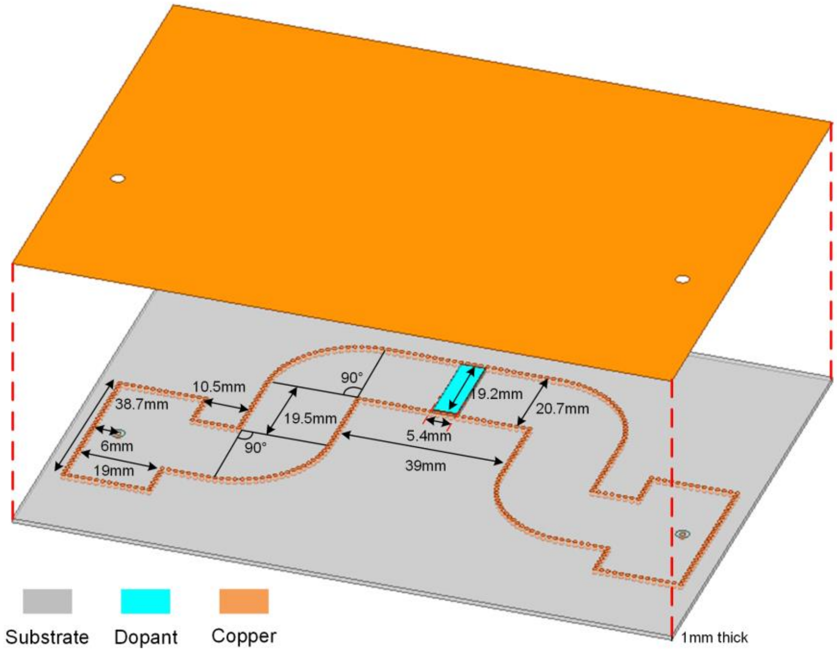

Supplementary Figure 10 | Geometry of supercoupling in a bending waveguide. 3D view of the supercoupling in a bending waveguide, i.e., experimental setup in Fig. 5c in the main text. Parameter values are indicated in the Figure, and the parameters in the main text that slightly differ from the Figure are the results after the SIW equivalent. The entire structure is axially symmetric. Metallic vias with radius 1 mm is arranged with the separation of 1.5 mm. The substrate is made in a dielectric material with ε_r = 2.2, while the dopant is made in a dielectric ceramic with ε_r = 37.

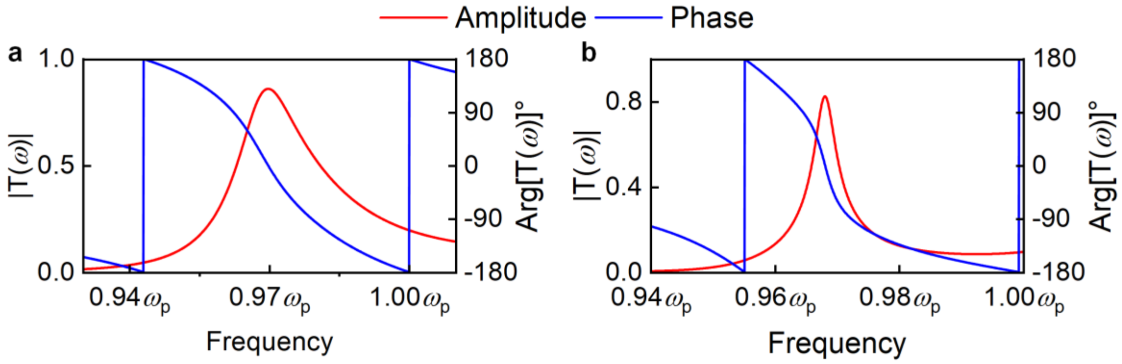

Supplementary Figure 11 | Simulated results of 3D configurations for the verified experimental setup for supercoupling features. (a) Simulated results of both transmission amplitude and phase (after calibration) in an asymmetric waveguide, i.e., Fig. 5a in the main text. **(b)** Simulated results of both transmission amplitude and phase (after calibration) in a bending waveguide, i.e., Fig. 5b in the main text.

Supplementary References

1. I. Liberal, A. M. Mahmoud, Y. Li, B. Edwards & N. Engheta. Photonic doping of epsilon-near-zero media. *Science*, **355**, 1058-1062 (2017).
2. Z. Zhou. et al. Substrate-integrated photonic doping for near-zero-index devices. *Nat. Commun.*, **10**, 4132 (2019).
3. J. D. Jackson. Classical Electrodynamics, 3rd ed. *Am. J. Phys.* **67**, 841-842 (1999).
4. Y. Li, I. Liberal & N. Engheta. Structural dispersion-based reduction of loss in epsilon-near-zero and surface plasmon polariton waves. Sci. Adv. 5, eaav3764 (2019).
5. Pozar, D. M. *Microwave Engineering* (John Wiley & Sons, New York, 2011).
